# Evolution and Conservation of Predicted Inclusion Membrane Proteins in Chlamydiae

**DOI:** 10.1155/2012/362104

**Published:** 2012-02-21

**Authors:** Erika I. Lutter, Craig Martens, Ted Hackstadt

**Affiliations:** ^1^Host-Parasite Interactions Section, Laboratory of Intracellular Parasites, Rocky Mountain Laboratories, National Institute of Allergy and Infectious Diseases, National Institutes of Health, Hamilton, MT 59840, USA; ^2^Research Technologies Branch, Rocky Mountain Laboratories, National Institute of Allergy and Infectious Diseases, National Institutes of Health, Hamilton, MT 59840, USA

## Abstract

*Chlamydia* spp. are obligate intracellular pathogens that replicate within a vacuole termed the inclusion. Chlamydiae extensively modify the inclusion membrane via the insertion of chlamydial inclusion membrane proteins (Incs) which decorate the cytosolic face of the inclusion. We have assessed the overall relatedness and phylogeny of Incs in order to identify potential evolutionary trends. Despite a high degree of conservation among Incs within *C. trachomatis* serovars, phylogenetic analysis showed that some Incs cluster according to clinical groupings suggesting that certain Incs may contribute to tissue tropism. Bioinformatic predictions identified Incs in five chlamydial species: 55 in *C. trachomatis*, 68 in *C. felis*, 92 in *C. pneumoniae*, 79 in *C. caviae*, and 54 in *C. muridarum*. Inc homologues were compared between chlamydial species and 23 core Incs were identified as shared among all species. Genomic expansion of Incs was identified in *C. pneumoniae, C. caviae*, and *C. felis* but not *C. trachomatis* or *C. muridarum*.

## 1. Introduction

 Chlamydiae are obligate intracellular pathogens that cause a variety of human and veterinary infections. *Chlamydia trachomatis* is the leading cause of preventable blindness worldwide and the most common bacterial sexually transmitted infection [1]. The species is comprised of 15 serovars that are associated with a wide spectrum of disease states including endemic trachoma (serotypes A to C), sexually transmitted infections (serotypes D to K), and a highly invasive granulomatous disease, lymphogranuloma venereum (LGV; serotypes L1 to L3) [1]. *C. pneumoniae* is a common cause of community acquired pneumonia and bronchitis [2] and has been linked to a spectrum of chronic diseases including atherosclerotic cardiovascular disease [3]. *C. felis* is a causative agent of feline chlamydiosis [4]. *C. caviae* and *C. muridarum *cause infections in guinea pigs and mice, respectively [5].

 Despite the differences in host tropism and disease, all *Chlamydia* spp. share several unique properties. Chlamydiae undergo a biphasic developmental cycle consisting of metabolically inactive infectious elementary bodies (EBs) and metabolically active noninfectious, reticulate bodies (RBs). Within the host cell, chlamydiae reside in a parasitophorous vacuole called the inclusion whose interactions with the host cell are unlike any other intracellular pathogen in that it is nonfusogenic with the endocytic pathway but intercepts exocytic vesicular traffic from the Golgi apparatus [6–8]. The inclusion membrane is at the interface between the pathogen and the host cell thus is situated to regulate exchange between the inclusion lumen and host cytosol [6, 9–13]. The inclusion membrane is heavily modified by the insertion of type III secreted chlamydial effector proteins shortly after the initiation of chlamydial protein synthesis. These inclusion membrane proteins, or Incs, are localized to the inclusion membrane and exposed to the host cytosol [14, 15].

 Much effort has been placed into the identification of Incs in chlamydiae through *in silico* predictions. While Incs share little sequence similarity to each other or known proteins in sequence databases thereby limiting speculation as to their function, they do however share a common secondary structural feature of a bilobed hydrophobic domain [16, 17]. The bilobed hydrophobic domain is a large hydrophobic region of 40 or more amino acids and may contain some centrally located hydrophilic residues to produce a characteristic bilobed hydropathy plot [16, 18]. This motif is largely specific to chlamydiae since comparative genomics only identified a few Inc-like open reading frames in other organisms [18]. This feature has been used to predict Inc proteins across different chlamydial species generating lists of putative proteins numbering from 36 to 59 for *C. trachomatis* [16, 18–21], 90 to 107 for *C. pneumoniae *[18, 19], 86 for *C. caviae *[19], 59 for *C. muridarum* [19], and 63 to 78 for *C. felis* [19, 22]. Despite the number of interactions with the host cell that are common throughout the genus and the potential for Incs to define interactions with the host cell at the interface of the inclusion and cytoplasm, there appears to be little conservation of Incs between species.

 Here we examine evolutionary relationships of Incs within *C. trachomatis *and between species in an effort to identify those Incs which might regulate conserved functions. The results demonstrate that overall there is a high level of conservation of Incs among *C. trachomatis *serovars at both the nucleotide and amino acid levels. Despite this overall high degree of similarity, certain Incs within *C. trachomatis *appeared to be evolving according to tissue tropism. A comparative genomics approach was used to identify Inc homologues shared or unique to *C. trachomatis*, *C. muridarum*, *C. felis*, *C. caviae,* and *C. pneumoniae*. Overall, there was little sequence conservation between distant homologues despite conservation in the hydrophobic nature and bilobed hydrophobic domains. Cross-genome comparisons identified a number of unique Incs to each species, shared Incs between paired species, and a core subset of Incs common to all species.

## 2. Materials and Methods

### 2.1. Inclusion Membrane Protein Prediction

 Inclusion membrane proteins were predicted in *C. trachomatis* L2/434/Bu (NC_010287.1), *C. felis *Fe/C-56 (NC_007899.1),* C. muridarum *Nigg (NC_002620.2), *C. caviae* GPIC (NC_003361.3) and *C. pneumoniae* AR39 (NC_002179.2). Kyte and Doolittle hydropathy plots [23] were generated for all proteins in the above genomes. The plots were scanned for the presence of 2 hydropathy peaks within 40 amino acids of each other or for the presence of one very large peak of greater than 40 amino acids. Added weight was given to proteins that were predicted to contain a transmembrane helix using TMHMM [24].

### 2.2. Identification of Inc Homologues

 For analysis of Incs between *C. trachomatis *strains, corresponding Incs were used from the following strains: A/Har-13 (NC_007429.1), B/Jali20/OT (NC_012686.1), B/TZ1A828/OT (NC_012687.1), D-LC (CP002054), D-EC (CP002052), D/UW-3/CX (NC_000117.1), E/11023 (CP001890), E/150 (CP001886), E/Sweden2 (FN652779), G/9301 (CP001930), G/9768 (CP001887), G/11074 (CP001889), G/11222 (CP001888), and L2b/UCH-1/proctitis (NC_010280.1). The resulting predicted Incs were then cross referenced with the other genomes using a PSI-BLAST to identify homologues [25].

### 2.3. Phylogenetic Distance and Genetic Divergence

 Nucleic acid sequence alignments for predicted Incs from different strains were generated using ClustalW [26]. Phylogenetic analysis of Incs was performed on nucleotide sequences using the Neighbor-Joining method [27] of MEGA4 [28]. Bootstrap consensus trees were inferred from 1000 replicates with the percentage of replicate trees in which the associated taxa clustered together in the bootstrap test displayed on the corresponding branches [29]. Estimates of evolutionary divergence were calculated using MEGA4. Results are based on the number of nucleic acid base pair substitutions per site on a pairwise analysis between all sequence pairs available for each* inc* and *pmp* using the Maximum Composite Likelihood Method [28, 30]. The mean genetic distance and pairwise comparisons were based on the number of nucleotide differences that included both transitions and transversions with gaps excluded. Additionally, the Nei-Gojobori Method [31] was performed comparing nonsynonymous (*d*_*N*_) and synonymous (*d*_*S*_) substitutions.

## 3. Results and Discussion

### 3.1. Genetic Divergence of Incs between *C. trachomatis* Strains

**Figure 1:**
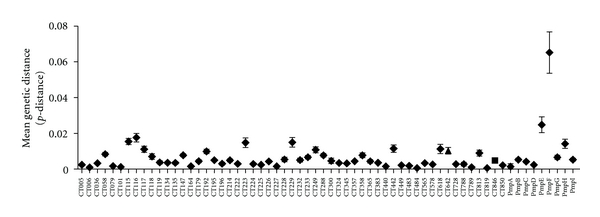
Genetic divergence of *inc*s and *pmp*s within *C. trachomatis. *Mean genetic distance within each predicted *inc* and *pmp *genes based on the average *p-*distance was determined from a pairwise comparison between all possible sequences for the same gene. Error bars represent mean with 95% confidence limit.

To identify putative Incs within *C. trachomatis *L2/434/Bu, a computational approach was designed to identify proteins that contained a hydrophobic domain of greater than 40 amino acids or two transmembrane domains of 20–30 amino acids separated by a small loop region [16, 19]. Each protein identified was analyzed using Kyte and Doolittle plot analysis to verify the presence of the characteristic bilobed hydrophobic domain [23]. A list of predicted Inc proteins is provided in Table 1. The Incs predicted by our computational method provided assemblages similar to those previously compiled [16, 19, 20]. Corresponding Incs from other *C. trachomatis *strains where complete genomes were available were downloaded and analyzed for evolutionary distance using MEGA4 (Figure 1). As an internal control for comparative purposes, the polymorphic outer membrane proteins (*pmpA-I*) were also analyzed using nucleotide sequences obtained from the available genomes. The mean genetic distances obtained for *C. trachomatis *Incs ranged from 0.001 (CT789) to 0.017 (CT116) with Incs CT115, CT116, CT223, and CT229 being the most divergent. Many of the Incs appeared genetically conserved in that they exhibited very little divergence (Figure 1). The genetic divergence seen among the *pmp*s was similar to what was previously described. *PmpE, pmpF*, and *pmpH *contained the most polymorphisms (mean genetic distances of 0.025, 0.065, and 0.014 resp.) [32]. These data suggest that despite being highly conserved, some Incs may be evolving at different rates, equivalent to the more divergent of the *pmp*s.

 Although distinct diseases and tissue tropisms are associated with different *C. trachomatis* serovars, the genomes examined to date exhibit a high degree of synteny and greater than 99% sequence identity [33–35]. The overall conservation seen between genomes indicates that there are relatively few loci involved in tissue tropism or that small polymorphisms in certain genes may greatly impact the infection process between serovars. Currently, there are few genetic loci that have been linked to clinical phenotype or tissue tropism within *C. trachomatis.* These include members of the Pmps, *pmpB, pmpC, pmpF, pmpG, pmpH *and *pmpI* [32, 36], *tarP* [37], *tox* [38, 39], *trpAB* [40–42], and *hctB* [43, 44]. Here we observed an overall conservation of Incs within different strains of *C. trachomatis* although four Incs (CT115, CT116, CT223, and CT229) appeared to be more divergent than others.

### 3.2. Phylogenetic Analysis of Incs within *C. trachomatis*

**Figure 2:**
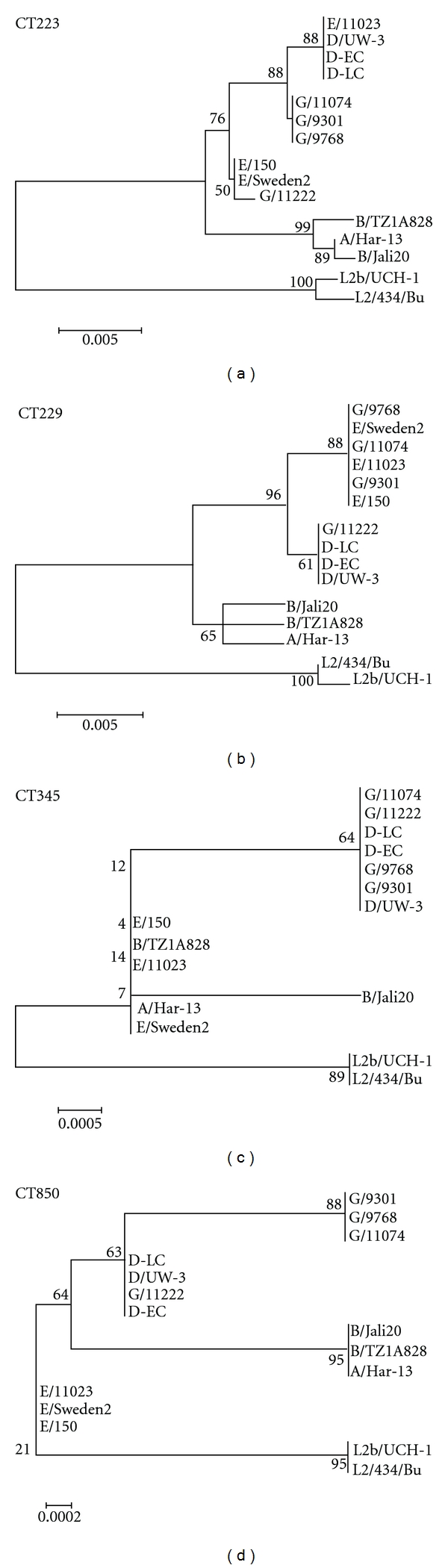
Phylogenetic reconstructions of *C. trachomatis Inc*s displaying tissue tropism clusters. Evolutionary history of Incs was inferred with the Neighbor-Joining method using the bootstrap test with 1000 replicates with the percentage of replicate trees associated with each clustered group shown next to the branches. Incs CT223 and CT229 cluster separate clades for ocular, genital, and LGV strains (a and b), CT345 clusters a separate LGV clade (c), and CT850 clusters separate LGV, ocular, and serovar E clades (d).

The variations in genetic divergence seen within predicted Incs of *C. trachomatis *suggested that certain Incs may be under different selective evolutionary pressures. Phylogenetic reconstructions of unrooted trees were performed for each Inc using existing genome sequences from all available serovars. The most divergent Incs, CT115, CT116, CT223, and CT229, exhibited clustering into clinical groupings with CT116 showing a separate clade for LGV strains while CT115, CT223, and CT229 exhibited separate clusters for genital, ocular, and LGV strains (Figures 2(a) and 2(b)). Incs CT214, CT383, CT618, and CT195 also demonstrated phylogenetic clustering according to clinical groupings although they showed less divergence based on genetic distance than Incs CT115, CT116, CT223, and CT229 (Figure 1). It was also possible to identify Incs that displayed partial clustering according to clinical groupings in that there were either separate clusters identified for ocular isolates or LGV isolates but not both. The most phylogenetically divergent clinical cluster was the LGV isolates. Separate LGV clusters could be identified for 35 Incs (Table 2, Figure 2(c)). Separate ocular clusters were identified for six Incs (Table 2, Figure 2(d)). Another trend that was evident was the separate clustering of serovar E isolates forming a separate clade from the ocular, LGV, and the other urogenital isolates. Finally, there were Incs that did not appear to segregate according to clinical disease (Table 2).

 The differences in phylogenetic clustering of Incs suggest that certain Incs may be evolving at different rates than others. Most of the Incs exhibited some clustering according to disease groupings. Seven Incs produced separate clusters for urogenital, LGV, and ocular strains, 35 Incs produced separate LGV clades, and 6 Incs produced separate ocular clades. These findings suggest the possibility that specific Incs may be evolving towards different infection strategies for different host tissues. A microarray analysis of niche specific genes previously identified four Incs, CT116, CT223, CT288, and CT618 as LGV specific [45] which were also identified in our study. However, no other Incs in that study were correlated with tissue tropism or clinical grouping.

 The greater phylogenetic divergence of those Incs producing separate LGV clades suggests that Incs within LGV strains may be undergoing evolutionary divergence at rates greater than those of other clinical groupings. This divergence has been noted with other phylogenetically defined tissue tropic genes including *tarP* [37] and the *pmp*s [32, 36]. Phylogenetic analysis of *tarP* indicated that the LGV isolates were the first to diverge to produce a distinct clade containing L1-L3 isolates [37]. A similar divergence of LGV strains was seen for the *pmp*s [32, 36]. This suggests that the evolutionary trend seen with the Incs in which LGV isolates appear more evolutionarily distinct coincides with other characterized genetic loci. It may be that LGV isolates in general show the greatest evolutionary divergence and that this divergence may not be limited to genes predicted to contribute to tissue tropism. The infections caused by LGV isolates differ from those caused by the ocular and urogenital serovars in that they are able to replicate within macrophages and cause a more invasive, systemic disease than the infections of mucosal epithelium caused by the ocular and urogenital strains [46]. The differences in host selective pressures may be driving the evolutionary differences seen within the LGV Incs as well as other loci.

### 3.3. Conservation of Incs between *Chlamydiae* Species

The same computational approach used for *C. trachomatis *was implemented to predict Incs for *C. caviae*, *C. felis*, *C. muridarum,* and *C. pneumoniae* (Table 1). All predicted Incs were cross-referenced to the other genomes using PSI-BLAST in attempts to identify divergent homologues in other species. Certain predicted Incs appear more than once in Table 1 due to potential similarity to more than one identified homologue. *C. pneumoniae *was predicted to contain the most Incs (92) with *C. felis *and *C. caviae *possessing 69 and 79, respectively. *C. trachomatis *and *C. muridarum *contained the fewest number of Incs within this comparison consisting of 55 and 54, respectively. Again, the predictions were highly overlapping but not identical to previous predictions [16, 18–22].

**Figure 3:**
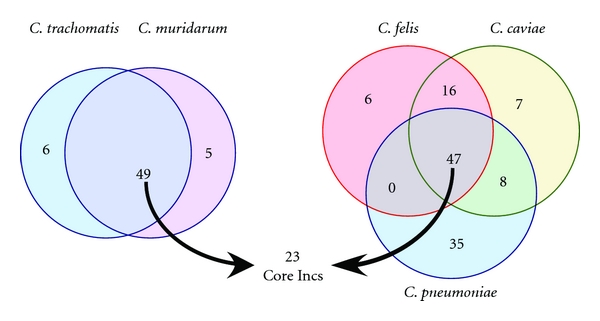
Venn diagram analysis of shared Inc homologues across Chlamydiae species. Two Venn diagrams are depicted showing shared Incs between *C. trachomatis *and *C. muridarum *(a) and between *C. felis, C. caviae*, and *C. pneumoniae* (b). Homologues that lacked an identifiable bilobed hydrophobic domain by Kyte and Doolittle analysis were not counted in the Venn diagram analysis. The numbers in the Venn diagram for *C. felis, C. caviae*, and *C. pneumoniae *total less than the total number of Incs defined for each species as only homologous sets of Incs were counted as one.

We were able to define a core subset of 23 Incs for which homologues could be identified in all five *Chlamydiae* species. *C. trachomatis *and *C. muridarum *were found to be the most related sharing 49 Inc homologues but also showed differences with each species containing unique Incs: 6 for *C. trachomatis *and 5 for *C. muridarum* (Figure 3, Table 1). The three remaining species, *C. felis*, *C. caviae,* and *C. pneumoniae*, also appeared very similar in that they shared a core of 47 Inc homologues. A pairwise comparison of these latter three species also identified shared Incs between any two given species with *C. felis *and *C. caviae *sharing a minimum 16 Incs, *C. caviae *and *C. pneumoniae *sharing 8, and *C. felis *and *C. pneumoniae *not sharing any outside of the core Incs (Figure 3, Table 1). Incs that appeared to be expanded in that there were more than one homologue per genome were only counted once in the Venn diagram. Genomic comparisons between all five species identified a core family of 23 conserved Incs for which Inc homologues are present in all species (CT005, CT006, CT058, CT134, CT179, CT195, CT232, CT233, CT288, CT324, CT365, CT383, CT440, CT449, CT483, CT484, CT565, CT616, CT618, CT642, CT728, CT788, and CT850) (Figure 3, Table 1). The analysis also identified Incs that were unique to each species (Table 1).

 Generally, if an Inc was identified in one species, then its homologues in other species were also identified as Incs, although some exceptions were noted (Table 1). *C. pneumoniae *CP0481 was identified as an Inc based on the presence of a bilobed hydrophobic domain whereas its homologues in *C. caviae *(CCA00586) and *C. felis *(CF0422) lack the characteristic bilobed hydrophobic domain. *C. pneumoniae *also encodes CP0667 which lacks a bilobed hydrophobic domain but contains homologues to CP0667 that do contain the hydrophobic domain (CP0387, CP0388, and CP0390; Table 1). Homologues to CP0667 in all four other chlamydial species also contain the characteristic bilobed hydrophobic domain (Table 1). *C. trachomatis *contained two genes which lacked a discernible bilobed hydrophobic domain; however the homologues of these genes in other chlamydial species are predicted to contain the characteristic hydrophobic bilobed domain (Table 1). As such, it appears that most Inc homologues maintain the bilobed hydrophobic domain.

**Figure 4:**
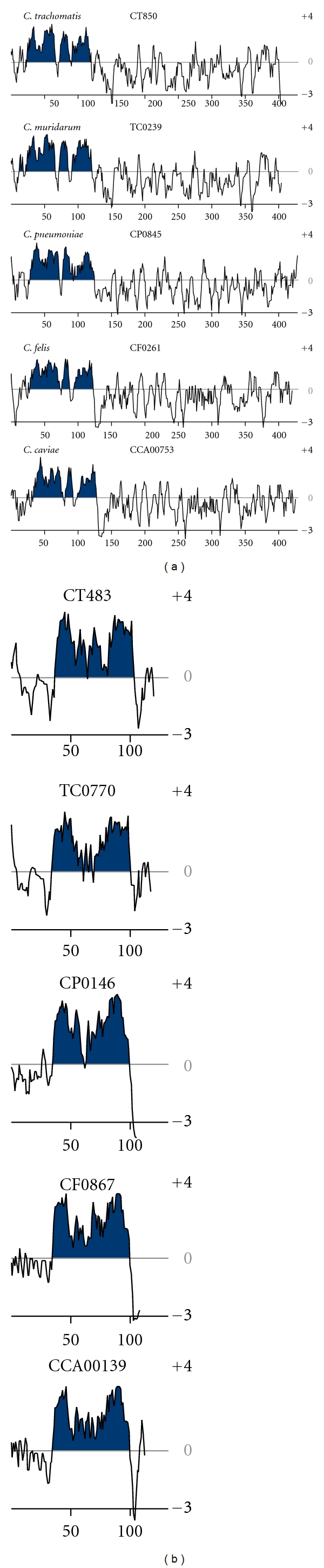
Hydropathy plot analysis and conservation of core Incs. Incs CT850 (a) and CT483 (b) from *C. trachomatis, C. muridarum, C. pneumoniae, C. felis*, and *C. caviae *were visualized using Kyte and Doolittle hydropathy plots. Regions of the bilobed hydrophobic domain are shown shaded.

Comparison of Incs between species provided an opportunity to evaluate the overall topology and conservation of Incs. Each Inc and its corresponding homologues identified were evaluated for the presence or absence of the characteristic bilobed hydrophobic domain. It was found that homologues of most Incs also contained a bilobed hydrophobic domain and that those homologues were also identified as Incs within this study suggesting that an Inc in one species has a corresponding homologue that is also an Inc in another species. Interestingly, the overall hydrophobic topology of the homologues was maintained despite a great degree of sequence divergence (an overall conserved identity of 27.3% for CT483 and 17.2% for CT850 at the amino acid level).  Figure 4 illustrates two examples of Inc homologues that were identified in all five species, CT850 (a) and CT483 (b). Not only the presence of the bilobed hydrophobic domain but also its location was conserved suggesting that the bilobed hydrophobic domain is an integral part of an Inc protein.

 Examination of all chlamydial species for which sequenced genomes were available indicates that the predicted Incs within each species represent a significant fraction of the genome. Overall, a great diversity of Incs was identified, not only those which are shared between species but also those which appeared to be unique to each individual species. It is possible that Incs present in only one species may play a role in infection that is unique to that species. There were also 23 Incs identified in all five species. These Incs represent core Incs that may be involved in conserved interactions with the host cell.

Very few of the known Incs have had functions assigned. IncA, first identified in *C. caviae* [47], is required for the homotypic fusion of inclusions in cells multiply infected with *C. trachomatis* [14] and when transfected into host cells blocks *C. caviae* development [48]. IncA appears to be nonessential for *C. trachomatis* survival and multiplication since clinical isolates of *C. trachomatis* lacking IncA have been isolated from patients [49]. IncA has been shown to have structural similarities to SNARE (soluble NSF (N-ethylmaleimide-sensitive factor) attachment protein receptors) proteins, a class of membrane proteins that control the specificity of vesicle fusion [50]. IncA has been shown to interact with the SNARE proteins Vamp3, Vamp7, and Vamp8 although depletion of these three SNAREs by siRNA had no deleterious impact on chlamydial growth [50]. A number of Rab-family GTPases are recruited to the chlamydial inclusion membrane in a species-dependent manner [51] and it appears that certain Inc proteins may play a role in specific Rab recruitment to the inclusion membrane. *C. trachomatis *CT229 mediates recruitment of Rab 4 [52] and *C. pneumoniae* Cpn585 displays affinity for Rab 1, Rab 10, and Rab 11 [53]. Other Incs known to recruit host proteins include IncG, which recruits the adaptor molecule 14-3-3*β* in a species-specific fashion [13]. Although a few Inc functions and interactions with host components have been identified, they are for the most part restricted to unique chlamydial species. One possibility is that the function of the majority of Incs and a reason for their duplication and diversity may not necessarily involve specific interactions with the host cell but be related to their unique hydrophobic structure and potential roles in the structural integrity of the inclusion membrane.

### 3.4. Genetic Expansion of Incs within Different *Chlamydiae* Strains

**Figure 5:**
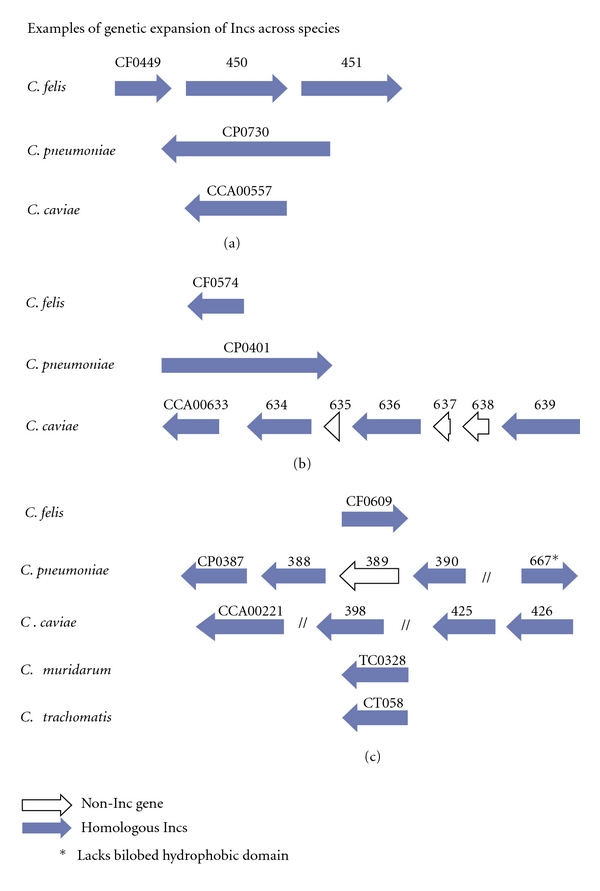
Examples of Inc expansion in *C. caviae, C. felis*, and *C. pneumoniae*. Three loci are depicted showing Inc expansion in *C. felis *(a), *C. caviae *(b), and *C. caviae *and *C. pneumoniae* (b and c). *designates that the predicted gene products lack the bilobed hydrophobic domain.

The *C. trachomatis *and *C. muridarum *genomes contained operons or clusters of Incs in which there was little sequence similarity between Inc open reading frames (CT115-119; CT222-229, Table 1). Although these Incs cluster together in the genome, they appear to have arisen independently of each other or may represent expansion of an ancestral *inc* that diverged at a rapid rate. In contrast, *C. felis*, *C. pneumonia, *and *C. caviae *each contained Inc operons in which there were intracluster similarities suggesting gene expansion within these genomes (Figure 5(c), Table 1). Additionally, orthologous Incs could be located outside of operons in other regions of the genome. Multiple examples of duplicated Incs could be identified in *C. felis, C. pneumonia,* and *C. caviae* however none were identified in *C. trachomatis *or *C. muridarum *(Table 1). *C. felis* contained three examples of Inc expansion, one of which is illustrated in Figure 5(a). The operon containing Incs CF0449-CF0451 had intracluster similarity but only a single identifiable homologue in either *C. caviae* (CCA00557) or *C. pneumoniae *(CP0730) (Figure 5(a)). *C. caviae *contained three genetic expansions of Incs, two of which are depicted in Figures 5(b) and 5(c). One Inc cluster (CCA00633–CCA00639) contained three non-Inc genes within the operon and was homologues to only one Inc in *C. felis *(CF0574) or *C. pneumoniae* (CP0401) (Figure 5(b)). The second Inc expansion (CCA00425, CCA00426, CCA00221, and CCA00398) corresponded to an Inc expansion in *C. pneumoniae *(CP0397-CP0390; CP0667), where one homologue, CP0667, did not contain an identifiable bilobed hydrophobic domain (Figure 5(c)). *C. pneumoniae *not only contained the greatest number of Incs but also exhibited the most Inc expansions (eight) of the species examined, one of which is depicted in Figure 5(c).

 The advent of genome sequencing and comparative genomics has led to the recognition that there has been genetic expansion of genes from *C. pneumoniae* [54, 55], *C. caviae *[56] but not *C. felis*. Initial comparisons between *C. pneumoniae *and *C. trachomatis *genomes showed a high degree of sequence conservation and synteny but also noted a remarkable increase in the number of predicted Pmp genes. *C. pneumoniae *encodes for 21 Pmps while *C. trachomatis *encodes for only 9 [54]. The amplification of the Pmp family of proteins suggests that either *C. pneumoniae *contains mechanisms that enhance genetic expansion and diversity or, alternatively, the unique biology of *C. pneumoniae* may have selected for these expansions. Similarly, we were able to identify Incs that have been expanded in *C. pneumoniae, C. caviae, *and *C. felis*. The genetic expansion of CT058 homologues was one of the most prominent in our study with *C. caviae *and *C. pneumoniae *each having four homologues. The regions flanking these genes also show a high degree of conservation [55]. Incs unique to *C. pneumoniae *appear to have undergone genetic expansion. *C. pneumoniae *lacks an IncA homologue but encodes a closely related set of Inc paralogues that are predicted to contain an IncA domain [57]. This group of paralogous genes has previously been referred to as the CP1054 family and each of these is shown to contain the characteristic bilobed hydrophobic domain characteristic of Incs. The reason for the level of genetic expansion seen in *C. pneumoniae, C. caviae,* and *C. felis *is uncertain but may represent a level of redundancy that is advantageous for these species with regards to infection or transmission that is not necessary for *C. trachomatis *or *C. muridarum*.

 While the analyses here are focused upon the human and veterinary pathogens that comprise the genus *Chlamydia*, members of the so-called environmental chlamydia of the family *Parachlamydiaceae* also express proteins displaying the characteristic bilobed hydrophobic structure of chlamydial inclusion membrane proteins. The *Parachlamydiaceae* are obligate intracellular symbiotes of amoebae believed to be ancestral to the pathogenic *Chlamydia* and contain several of the virulence factors expressed in *Chlamydia*. The genomes of the environmental chlamydiae are larger; that of *Protochlamydia amoebophila* is approximately 2.41 Mbp [58] versus that of *C. trachomatis* serovar D which is 1.04 Mbp [34]. Despite the much larger genome, the number of putative Incs in *P. amoebophila* is only twenty-three [59]. Of these, only three (pc0156, pc0184, and pc1857) bear significant similarity to chlamydial Incs and the Incs to which they are most closely related are among the core Incs identified here in *Chlamydia spp.* Like the chlamydial inclusion membrane proteins, the functions of the Incs from *P. amoebophila* are largely unknown although five of these have been confirmed as localized to the inclusion membrane [59], and thus the predictive value of the bilobed hydrophobic domain appears to be viable in this family as well.

## 4. Conclusions

 The chlamydial inclusion is extensively modified very early in infection by the insertion of a family of type III secreted effector proteins collectively known as Incs. Once the inclusion membrane is modified by de novo synthesized chlamydial proteins, a number of interactions with the host cell are initiated [6, 7, 60–62]. The unique interactions of the chlamydial inclusion with the host cell and biological similarities between chlamydial species would lead one to predict that pathogen proteins situated to potentially influence interactions might be conserved among chlamydiae. The Inc genes are, however, among the most variable between chlamydial genomes with only a relatively small number of orthologs conserved in all species [19]. Instead, each chlamydial species contains a number of unique Incs. A comparative genomics approach coupled with phylogenetic analysis was therefore applied to predicted Inc proteins of *C. trachomatis, C. muridarum, C. caviae, C. felis,* and *C. pneumoniae* in an effort to identify those Incs that might contribute to conserved functions. Using this approach, a core set of 23 Incs was identified.

 Taken together, our data suggest that there is a high degree of conservation of Inc proteins within serovars of *C. trachomatis* but that specific Incs show evidence of evolutionary divergence that phylogenetically separate certain Incs into clinical clusters (LGV, ocular, and urogenital). By taking a comparative genomics approach, a core set of Incs were identified which are common to all five species examined. The core Inc genes identified may represent proteins involved in conserved interactions between the chlamydia and host. Incs unique to each species were also identified. The diversification of Incs between species suggests that certain Incs may have evolved unique pathogenic roles within these species. A more complete understanding of the interactions of the Inc proteins may provide for new insights into chlamydial pathogenesis.

## Figures and Tables

**Table 1:** Open reading frames with predicted Inc-like domains^a^.

*C. trachomatis*	*C. muridarum*	*C. caviae *	*C. felis*	*C. pneumoniae*
—^b^	—	CCA00222	CF0784	CP0230
—	TC0328	CCA00221	CF0785	CP0229
—	—	CCA00353	—	CP0314
CT005	TC0273	CCA00290	CF0713	CP0310
CT006	TC0274	CCA00291	CF0711	CP0311
—	—	CCA00318	—	—
CT036	TC0306	CCA00647	—	CP0642
—	—	CCA00645	—	—
—	—	CCA00361	CF0646	CP0322
—	—	—	CF0611	—
—	—	CCA00397	CF0610	—
CT058	TC0328	CCA00426/425/398/221	CF0609	CP0387/388/390/667*
CT079	TC0351	CCA00449	—	CP0424
CT101	—	CCA00470	CF0537	CP0446
CT115^†^	TC0391	—	—	—
CT115	—	CCA00622	CF0382^†^	—
CT116	TC0392	—	—	—
CT117	TC0393	—	—	—
CT118	TC0394	—	—	—
—	—	CCA00636/633/634/639	CF0574	CP0401^†^
—	—	CCA00530	CF0479/369	CP0404/407
—	—	CCA00434	CF0574^†^	CP0405/404
CT119^†^	TC0396	CCA00550	CF0458	—
—	—	CCA00550	—	CP0581^†^
CT134	TC0411	CCA00537	CF0471	CP0520/519
CT135	TC0412^†^	CCA00538	—	CP0522*
CT135	TC0412	CCA00538^†^	CF0470	CP0521*/522*
CT147	TC0424	CCA00616	CF0388	CP0623
—	—	CCA00621	CF0383	CP0627
—	—	CCA00620	CF0384	—
—	—	CCA00619	CF0385	CP0626
CT164	—	—	—	—
—	—	CCA00557	CF0449/450/451	CP0730^†^
—	—	CCA00557^†^	CF0449/450/451	—
		CCA00576	CF0425	CP0595
CT179	TC0451	CCA00591	CF0412	CP0534
CT192	TC0464	—	—	—
CT195	TC0468	CCA00494	CF0513	CP0470
—	—	CCA00514	CF0493	CP0493/495
CT196	TC0469	—	—	—
CT214	TC0486	CCA00500	CF0508	—
—	—	—	CF0504	—
—	—	CCA00513	—	CP0707^†^
—	—	CCA00513^†^	CF0494^†^	CP0730
CT222	—	—	—	—
CT223	TC0495	—	—	CP0709
CT224	—	—	—	—
CT225	—	—	—	—
—	TC0496	—	—	CP0390
CT226	TC0497	—	—	—
CT227	TC0498	—	—	—
CT228	TC0499^†^	CCA00513	—	—
CT229	TC0500	—	—	—
CT232	TC0503	CCA00491	CF0516	CP0467
CT233	TC0504	CCA00490	CF0517	CP0466
CT244*	TC0515	—	CF0528*	CP0455*
CT249	TC0520	—	—	—
—	—	CCA00474	CF0533	—
—	—	CCA00497	—	CP0473
—	—	CCA00586*	CF0422*	CP0481
—	—	CCA00513	—	CP0539
—	—	CCA00582*	CF0418	CP0581
—	—	CCA00576	CF0425	CP0595
—	—	CCA00430	—	—
—	—	CCA00424	CF0583	CP0649
—	—	CCA00398	CF0609	CP0387
—	—	CCA00397	CF0610	—
—	—	CCA00352	—	CP0313
CT288	TC0561	CCA00351	CF0654	CP0709^†^
—	TC0573	—	—	—
CT300	TC0574	—	—	—
CT324	TC0598	CCA00700	CF0311	CP0703
CT345	TC0624	—	—	—
CT357	TC0636	—	—	—
CT358	TC0637	—	CF0218	—
—	—	CCA00361	CF0646	CP0322
—	—	CCA00360	CF0647	CP0321
—	—	CCA00334/00339	CF667/668/669	—
—	—	CCA00325	CF0677/678	CP0742
—	—	CCA00318	CF0685	—
CT365	TC0644	CCA00269	CF0739	CP0280
CT383	TC0662	CCA00263	CF0745	CP0274
—	—	CCA00156	CF0851	CP0825/163
CT440	TC0724	CCA00188	CF0818	CP0198
CT442	TC0726	CCA00186	—	CP0196
CT449	TC0734	CCA00177	CF0830^b^	CP0185
CT449^†^	TC0734	—	—	CP0185
CT483	TC0770	CCA00139	CF0867	CP0146
CT484	TC0771	CCA00138	CF0868	CP0145
—	—	CCA00156	CF0851	CP0163
—	—	—	CF0048	—
CT565	TC0854	CCA00941	CF0073	CP1049
CT616*	TC0906*	CCA01002	CF0010	CP1117
CT618	TC0908	CCA01004	CF0008	CP1119
CT642	TC0010	CCA00987	CF0026	CP1102
—	TC0011	—	—	—
CT728	TC0101	CCA00898	CF0116	CP1000
—	—	—	CF0128	—
CT788	TC0171	CCA00832	CF0182	CP0923
CT789	—	—	—	—
CT813	TC0199	—	—	—
—	—	CCA00801	CF0215	—
—	—	CCA00800	CF0216	—
—	—	CCA00799	CF0217	—
—	—	CCA00797	CF0218	—
—		CCA00795	CF0219	—
—	—	CCA00794	CF0220	—
—	—	CCA00793	—	—
CT819	TC0206	CCA00786	CF0227	CP0890*
CT846	TC0234	CCA00758*	CF0256	CP0850*
CT850	TC0239	CCA00753	CF0261	CP0845
—	—	CCA00733	CF0283	CP0823
—	—	CCA00708	—	—
—	—	CCA00702	—	—
—	—	—	—	CP0236
—	—	—	—	CP0381
—	—	—	—	CP0385
—	—	—	—	CP0386
—	—	—	—	CP0391
—	—	—	—	CP0392
—	—	—	—	CP0396
—	—	—	—	CP0450
—	—	—	—	CP0474
—	—	—	—	CP0544
—	—	—	—	CP0547
—	—	—	—	CP0549
—	—	—	—	CP0550
—	—	—	—	CP0551
—	—	—	—	CP0553
—	—	—	—	CP0554
—	—	—	—	CP0597
—	—	—	—	CP0602
—	—	—	—	CP0605
—	—	—	—	CP0607
—	—	CCA0062*	—	CP0627
—	—	—	—	CP0640
—	—	—	—	CP0641
—	—	—	—	CP0646
—	—	CCA00513	—	CP0649/797
—	—	CCA00674	CF0337	CP0675
—	—	—	—	CP0703
—	—	—	—	CP0733
—	—	—	—	CP0750
—	—	—	—	CP0763
—	—	—	—	CP0728/764/766/769
—	—	—	—	CP0795
—	—	—	—	CP0801
—	—	—	—	CP0935

^a^Data from Incs identified here and in [16, 18–21].

^b^indicates the absence of an identifiable homologue identified by PSI-BLAST.

^c∗^indicates that a homologue lacks the characteristic bilobed hydrophobic domain when analyzed with Kyte and Doolittle plots

^d†^indicates Inc used in PSI-BLAST.

**Table 2:** Phylogenetic clustering of Incs in *C. trachomatis*.

Ocular, genital, and LGV	CT115, CT223, CT214, CT229, CT383, CT618, CT195
LGV specific	CT036, CT058, CT101, CT116, CT117, CT118, CT119, CT134, CT135, CT147, CT164, CT192, CT196, CT228, CT232, CT233, CT249, CT288, CT300, CT324, CT345, CT357, CT358, CT365, CT383, CT442, CT449, CT484, CT578, CT642, CT728, CT788, CT789, CT813,CT846

Ocular specific	CT224, CT225, CT226, CT383, CT846, CT850

No correlation with disease	CT005, CT006, CT079, CT227, CT440, CT565

Serovar E specific clades	CT058, CT116, CT117, CT192, CT233, CT249, CT288, CT324, CT357, CT358, CT365, CT383, CT442, CT483, CT578, CT642, CT846, CT850
